# Efficacy of post-exercise recovery strategies for elite soccer players: a network meta-analysis

**DOI:** 10.3389/fphys.2026.1760392

**Published:** 2026-01-21

**Authors:** Jie Liu, Qiang Li, Yu Han

**Affiliations:** 1 School of Sports and Training, Tianjin University of Sport, Tianjin, China; 2 School of Physical Education, Tianjin University of Sport, Tianjin, China; 3 School of Social Sports, Tianjin University of Sport, Tianjin, China

**Keywords:** elite soccer, network meta-analysis, post-exercise, post-match, recovery strategy

## Abstract

**Objective:**

This study aimed to systematically compare the efficacy of various recovery strategies for improving neuromuscular function, muscle damage, and subjective fatigue in elite soccer players following matches or validated simulations, and to provide evidence-based guidance for clinical practice.

**Methods:**

Following PRISMA-NMA guidelines, we systematically searched PubMed, Embase, Cochrane Library, Web of Science, and Scopus for randomized controlled trials evaluating post-match or post-simulation recovery strategies in professional and semi-professional soccer players. A Bayesian random-effects model was applied to conduct the network meta-analysis. Effect sizes were reported as mean differences (MD) with 95% credible intervals (CrI), and intervention efficacy was quantified using Surface Under the Cumulative Ranking (SUCRA) values.

**Results:**

Twenty-three RCTs involving 388 participants and 17 recovery interventions were included. Key findings were as follows: (1) Far-infrared therapy (FIR) was most effective in improving Countermovement jump (CMJ) height (SUCRA = 98.3%); (2) Intermittent negative pressure therapy (INPT) produced the greatest reduction in Creatine kinase (CK) levels (SUCRA = 91.0%); (3) Portable cold compression therapy (PCMcold) had the strongest effect on alleviating Muscle soreness (MS) (SUCRA = 98.9%); (4) FIR and Intermittent vascular occlusion (IVO) significantly improved Maximal voluntary contraction (MVC), although Hyperoxic gas (Hyp) ranked highest (SUCRA = 89.8%); and (5) no intervention significantly improved 20-m sprint performance (all 95% CrI included zero), and although IVO ranked first (SUCRA = 84.5%), its evidence reliability was low.

**Conclusion:**

Personalized post-match recovery in elite soccer should be based on specific targets. FIR is recommended for CMJ restoration, INPT for muscle damage repair, PCMcold for reducing subjective soreness, and FIR may be considered for MVC recovery. No superior intervention was identified for 20-m sprint recovery; therefore, basic recovery measures are advised. Future multi-arm RCTs are required to validate combined recovery strategies and standardize intervention parameters.

## Introduction

Soccer is a paradigmatic high-intensity intermittent sport characterized by frequent sprints, jumps, tackles, rapid directional changes, and substantial metabolic demands (Stølen et al., 2005). The combined effect of these factors imposes multi-dimensional physiological stress on athletes (Nédélec et al., 2012). Unlike single-task laboratory assessments, soccer competitions involve fluctuating intensity, complex technical and tactical requirements, and variable movement patterns. These demands trigger pronounced physiological responses, including muscle damage and neuromuscular fatigue that can lead to transient declines in essential performance indicators such as lower-limb power and acceleration capacity, thereby influencing subsequent match outcomes (Bush et al., 2015; Markus et al., 2021; Querido et al., 2022). For elite players, this challenge is intensified by dense competition schedules (Nédélec et al., 2012). Empirical data show that elite players participate in an average of 5.5 matches per month, with only 3–4 days of recovery between games (Nédélec et al., 2012), a timeframe considerably shorter than that required for complete physiological restoration. Objective evidence confirms that such congested schedules exert substantial adverse effects on match performance and recovery outcomes (Stølen et al., 2005): Performance decline: After three consecutive matches with < 72 h of recovery, elite players exhibit a 12%–15% reduction in high-intensity sprint distance and a 8%–10% decrease in successful passing accuracy (Bush et al., 2015; Nédélec et al., 2012) Impaired recovery: Short recovery intervals (<48 h) lead to a >30% elevation in muscle damage markers (e.g., CK) and an 8%–10% decline in neuromuscular function compared with adequate recovery (>7 days) (Nédélec et al., 2012; Bush et al., 2015) Increased injury risk: Players with ≥2 matches per week have a 2.3-fold higher risk of overuse injuries than those with ≤1 match per week, primarily due to incomplete recovery of muscle microdamage and cumulative fatigue (Gabbett and Jenkins, 2011). Consequently, rapid and effective post-match recovery is not merely a supplementary component of training but a critical determinant of training continuity, performance stability, and long-term athlete health. Notably, it is important to acknowledge that most targeted recovery interventions yield modest or incremental benefits, as fundamental factors such as sufficient sleep and balanced nutrition often play a more pivotal role in recovery than any single specialized intervention. Inadequate recovery not only impairs subsequent performance but also increases the risk of overuse injuries and cumulative fatigue (Nédélec et al., 2012; Gabbett and Jenkins, 2011), underscoring the need for evidence-based recovery strategies tailored to the specific demands of soccer.

Over the past decade, a wide range of recovery interventions for professional soccer players has been investigated, including physical therapies cold-water immersion, (CWI) (Babak et al., 2021; Coelho et al., 2021), foam rolling (Rahimi et al., 2020; Bibić et al., 2025), cryotherapy (Clifford et al., 2018), massage (Hongsuwan et al., 2015), compression therapy (Marqués-Jiménez et al., 2018; Gimenes et al., 2021), and electrical stimulation (Gonçalves et al., 2024), nutritional supplementation (Abreu et al., 2023), and active or passive recovery protocols (Pooley et al., 2020). However, a major limitation of existing research is its heavy reliance on single-task laboratory tests that fail to capture the multifactorial physiological and locomotor demands of match play. Even soccer-specific laboratory protocols exhibit limited ecological validity. For example, athletes performing the Loughborough Intermittent Shuttle Test (LIST), a protocol designed to simulate soccer, displayed alterations in muscle-damage markers due to frequent accelerations, decelerations, and changes of direction (Magalhães et al., 2009). Although these responses resembled those observed after official matches, the findings nonetheless highlight that even “soccer-specific” simulations cannot fully replicate the complex fatigue profiles induced by competition, let alone isolated strength or running tests. As a result, outcomes derived from such laboratory studies cannot be reliably extrapolated to real post-match scenarios (Nédélec et al., 2012). Notably, match-induced fatigue varies substantially: positional differences in running volume, individual physiological responses to tackles, and match intensity (e.g., derbies vs. regular games) lead to heterogeneous fatigue profiles and recovery trajectories (Nicholas et al., 2000; Stone et al., 2011; Robineau et al., 2012). This often-overlooked heterogeneity complicates generic recovery strategies, as interventions effective for one player or context may not apply to another. This misalignment between laboratory research and on-field demands has left coaches and sport scientists uncertain about which recovery strategies most effectively mitigate soccer-specific fatigue.

To address this gap, the present study focuses exclusively on recovery strategies applied after official soccer matches or validated simulation protocols that reproduce the technical, tactical, and load characteristics of competition to the greatest extent possible. Given the paucity of relevant studies to date, this approach is also intended to maximize the sample size, thereby ensuring the scientific rigor of the study findings. This targeted design is strengthened by the use of network meta-analysis (NMA), an advanced statistical method that overcomes the limitations of traditional pairwise meta-analysis. By integrating direct and indirect comparisons, NMA allows for the simultaneous evaluation of multiple interventions and provides a hierarchical ranking of their relative efficacy (Salanti et al., 2011). Although NMA has been increasingly applied in sports science to assess training and injury-prevention strategies, its use in evaluating recovery interventions for elite soccer remains limited, particularly in studies addressing match-specific or match-simulation fatigue (Robles-Palazón et al., 2024). Moreover, previous NMAs have often employed narrow inclusion criteria (e.g., focusing solely on professional athletes) or limited outcome domains, thereby excluding broader elite populations such as semi-professional players and failing to assess comprehensive recovery markers.

Based on these gaps, the present NMA aims to (Stølen et al., 2005): systematically compare the efficacy of current recovery strategies used by elite soccer players (professional and semi-professional) following matches or validated simulations (Nédélec et al., 2012); quantify the relative effectiveness of these strategies across key indicators of neuromuscular function, muscle damage, and subjective fatigue, including countermovement jump (CMJ), creatine kinase (CK), 20-m sprint, maximal voluntary contraction (MVC), and muscle soreness (MS); and (Bush et al., 2015) provide evidence-based recommendations to support clinical decision-making and guide future research.

## Materials and methods

This systematic review and NMA was prospectively registered in the International Prospective Register of Systematic Reviews (PROSPERO), under registration number: CRD420251240850, and the study protocol underwent peer review and was published in a scientific journal. All procedures were conducted in full accordance with the Preferred Reporting Items for Systematic Reviews and Meta-Analyses extension for Network Meta-Analyses (PRISMA-NMA) guidelines.

### Search strategy

Literature retrieval was conducted by the research team for studies published from inception to 13 November 2025 across five major international electronic databases: PubMed, EMBASE, the Cochrane Central Register of Controlled Trials (CENTRAL), Web of Science, and Scopus. The search strategy followed the PICOS framework: (P) Population: elite soccer players, including professional and semi-professional athletes; (I) Interventions: various recovery modalities such as active recovery, blood flow restriction training, cold-water immersion, contrast water therapy, compression garments, active relaxation training, cryotherapy, cold clothing wear, sleep or daytime napping, pneumatic cooling, foam rolling, mindfulness interventions, nutritional supplementation, static stretching, neuromuscular recovery training, massage, and electrical stimulation; (C) Comparators: control conditions without specific recovery effects (e.g., passive recovery or placebo interventions lacking physiological recovery efficacy); (O) Outcomes: CMJ, CK, 20-m sprint performance, MVC and MS; and (S) Study design: Randomized controlled trials (RCT). Detailed search strategies for each database are presented in Supplementary Material S1. Additionally, hand searching was performed by screening the reference lists of relevant systematic reviews and meta-analyses to identify further eligible studies.

### Inclusion criteria

Studies were included if they (Stølen et al., 2005): were published in peer-reviewed scientific journals (Nédélec et al., 2012); involved adult male or female soccer players (≥18 years old) (Bush et al., 2015); focused on elite players (professional or semi-professional); The elite soccer players in this study refer to professional (full-time contracts, top/second-tier leagues) and semi-professional (part-time contracts/registered in semi-professional club, regional/lower-tier leagues) athletes with structured training and competitive experience, excluding high-level amateur players (Clifford et al., 2018; Alexander et al., 2022; Andersson et al., 2008; Bouzid et al., 2018). Markus et al. (2021) examined recovery interventions administered after fatigue induced by official soccer matches or validated simulation protocols (Querido et al., 2022); focused on match or LIST-induced fatigue in elite football players; and (Gabbett and Jenkins, 2011) allocated participants to at least two groups (one control group and one intervention group).

### Exclusion criteria

Studies were excluded if they (Stølen et al., 2005): involved athletes from sports other than soccer (Nédélec et al., 2012); included adolescent players (<18 years old) (Bush et al., 2015); induced fatigue using isolated exercise protocols (e.g., single running, strength training, or plyometric sessions, etc.) instead of matches or validated simulations (Markus et al., 2021); implemented recovery interventions before or during fatigue induction; or (Querido et al., 2022) were conducted in extreme environmental conditions (e.g., high altitude or high temperature).

### Study selection

Two researchers (JL and YH) independently screened and selected studies using EndNote reference management software. After removing duplicates, titles were screened to exclude non-RCTs, reviews, conference proceedings, study protocols, and letters. Abstracts of the remaining studies were then assessed to make preliminary inclusion or exclusion decisions. Full texts of potentially eligible studies were subsequently reviewed to confirm compliance with all predefined criteria. Throughout the process, the two researchers worked independently, and their decisions were cross-checked. Studies with consistent judgments were included directly, while disagreements were resolved through discussion with a third reviewer (QL) to reach consensus.

### Data extraction

A standardized, pretested data extraction form was used to collect and organize key study information. Extracted data included (Stølen et al., 2005): study authors (Nédélec et al., 2012); publication year (Bush et al., 2015); country (Markus et al., 2021); participant characteristics (sample size, age, competitive level) (Querido et al., 2022); fatigue-induction protocol (Gabbett and Jenkins, 2011); detailed intervention procedures and parameters (Babak et al., 2021); measurement time points; and (Coelho et al., 2021) outcome types and raw data. Data extraction was performed independently by two researchers (Jie Liu and Yu Han), with discrepancies resolved by consultation with a third researcher (Qiang Li). When studies did not directly report means and standard deviation (Brownstein et al., 2019), values were converted from medians, ranges, or interquartile ranges, or digitally extracted from bar or line graphs using WebPlotDigitizer (Version 4.7; San Francisco, California, United States; Salanti, 2012).

### Risk of bias assessment

Two researchers independently assessed the risk of bias using the Cochrane Collaboration’s Tool for Assessing Risk of Bias in Randomized Controlled Trials (version 5.2.0). Given the nature of recovery interventions, participant blinding was generally not feasible. Seven domains were evaluated (Stølen et al., 2005): random sequence generation (Nédélec et al., 2012); allocation concealment (Bush et al., 2015); blinding of participants (Markus et al., 2021); blinding of personnel (Querido et al., 2022); incomplete outcome data (Gabbett and Jenkins, 2011); selective reporting; and (Babak et al., 2021) other potential sources of bias. For studies that attempted to minimize performance bias, measures such as using intervention carriers with identical appearance or taste (e.g., creatine vs. maltodextrin powders, indistinguishable far-infrared devices), providing standardized information intended to mask differences between interventions (e.g., informing participants that placebo and active treatments have equivalent effects), or separating intervention providers from outcome assessors were documented.

### Data analysis

Data analysis was conducted using R software (version 4.5.1) and GeMTC (van Valkenhoef et al., 2012). The primary statistical procedures were performed under a Bayesian random-effects framework using the gemtc and rjags packages in R (Shim et al., 2019). Markov Chain Monte Carlo (MCMC) simulation was applied to estimate the posterior distributions of effect sizes for each intervention, and corresponding probability distribution characteristics were derived (Nagaraja and Braga-Neto, 2018; Mavridis and Salanti, 2013; Jansen et al., 2008). To ensure the robustness of findings, both consistency and inconsistency models were evaluated, and node-splitting analyses were performed for closed loops to assess local inconsistency between direct and indirect evidence (Bhatnagar et al., 2014). Convergence of MCMC chains was examined using the Brooks–Gelman–Rubin diagnostic alongside trace and density plots. Effect sizes were reported as mean differences (MD) with 95% credible interval (CrI), with statistical significance defined as CrI that did not include zero. Forest plots and league tables were used to summarize pairwise comparisons across all interventions. Treatment ranking probabilities and Surface Under the Cumulative Ranking (SUCRA) values were calculated to quantify the relative efficacy of interventions (Salanti et al., 2011; Trinquart et al., 2016), with higher SUCRA values indicating greater likelihood of superior performance. Network geometry was visualized using network plots, where nodes represented intervention types and edges indicated direct comparisons. Covariate significance in meta-regression analyses was determined by whether the 95% confidence interval of the regression coefficient excluded zero. Network plots and funnel plots were generated using Stata 15.0 to assess publication bias (Salanti et al., 2011), and cumulative ranking probability plots were produced using the ggplot2 package in R.

## Results

### Study identification and selection

**FIGURE 1:**
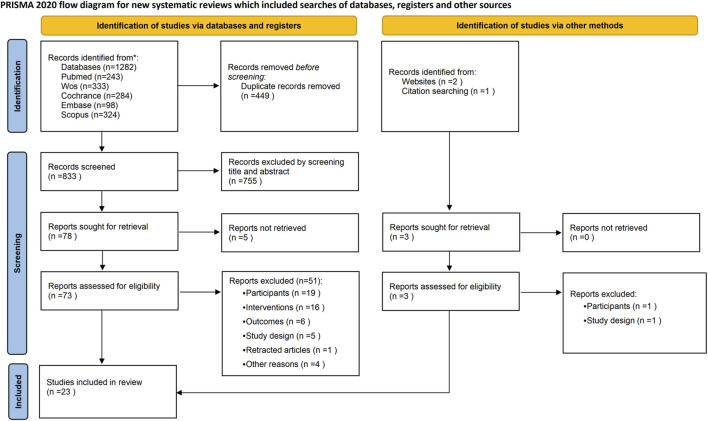
Flow diagram.

A total of 1,285 records were initially identified (1,282 through electronic databases and three through hand-searching). After removing 449 duplicates using EndNote, 836 studies were screened by title and abstract, resulting in the exclusion of 755 records. The remaining 81 full-text articles were assessed for eligibility, of which 58 were excluded for various reason. Ultimately, 23 studies met the inclusion criteria and were incorporated into the meta-analysis. The study selection process is illustrated in Figure 1.

### Characteristics of included studies

A total of 23 randomized controlled trials involving 388 participants were included. Interventions encompassed active recovery (ACT), cold-water immersion (CWI), cryotherapy (Cry), thermoneutral water therapy (TW), Phase change material cold (PCMcold), far-infrared therapy (FIR), intermittent negative pressure therapy (INPT), Hyperoxic gas (Hyp), among others. Nineteen studies (Clifford et al., 2018; Alexander et al., 2022; Andersson et al., 2008; Bouzid et al., 2018; Brownstein et al., 2019; Ascensão et al., 2011; Bouchiba et al., 2022; Cockburn et al., 2013; Daab et al., 2021; Daab et al., 2025; Fullagar et al., 2016; Goulart et al., 2021; Mujika et al., 2000; Nasser et al., 2023; Rey et al., 2012; Rey et al., 2019; Shiravand et al., 2024; Tessitore et al., 2008; Tseng et al., 2024) reported CMJ, twelve studies (Andersson et al., 2008; Bouzid et al., 2018; Ascensão et al., 2011; Bouchiba et al., 2022; Daab et al., 2021; Daab et al., 2025; Fullagar et al., 2016; Nasser et al., 2023; Tseng et al., 2024; Abedi et al., 2018; Maior et al., 2020; von Stengel et al., 2018) reported CK, nine studies (Andersson et al., 2008; Bouzid et al., 2018; Ascensão et al., 2011; Bouchiba et al., 2022; Daab et al., 2021; Daab et al., 2025; Goulart et al., 2021; Nasser et al., 2023; Rey et al., 2012) reported 20-m sprint performance, seven studies (Clifford et al., 2018; Bouzid et al., 2018; Brownstein et al., 2019; Ascensão et al., 2011; Bouchiba et al., 2022; Daab et al., 2021; Daab et al., 2025) reported MVC, and six studies (Clifford et al., 2018; Andersson et al., 2008; Bouzid et al., 2018; Daab et al., 2025; Tseng et al., 2024; Ezequiel et al., 2012) reported MS. Detailed study characteristics are provided in Table 1.

**TABLE 1:** Characteristics of included studies.

Author	Year	Country	Participants	Samplesize	Mean age	Exercise protocol	Recovery intervention	Time of measures	Outcomes
Abedi	2018	Iran	30 male professional players from second-highest division league in Iranian football league	CWI: 10 ACT: 10 CON: 10	22.4 ± 2.38 years	Simulated football match	Ex: 10 min CWI, 10 °C Ex2: ACT Ex3: PAS	48 h	CK
Alexander	2022	United Kingdom	20 elite male soccer players	Cryo: 10 CON: 10	18 ± 0.5 years	Training session: Including football-specific movements such as running and jumping, changing direction, and confrontation, etc.	Ex: Cryo-compression Ex2: PAS, resting in a semi-reclining position	Immediately	CMJ
Andersson	2008	Sweden	17 elite female soccer players	ACT: 8 CON: 9	22.07 ± 3.44 years	Football match	Ex: ACT Ex: PAS	Immediately	CMJ; MS 20-m sprint; CK
Ascensão	2011	Portugal	20 male from national league teams	CWI: 10 TWI: 10	18.2 + 1.39 years	Football match	Ex: 10 min CWI, 10 °C CON: 10 min TWI, 35 °C	24 h	CK; CMJ MVC 20-m sprint
Bouchiba	2022	Tunisia	12 male soccer players from the third Tunisian National league	CWI: 12 TWI: 12	22.9 ± 0.9 years	Simulated football match using the LIST	Ex: 10 min CWI, 10 °C ± 2 °C CON: 10 min TWI, 28 °C ± 2 °C	48 h	MVC; CMJ 20-m sprint CK
Bouzid	2018	Tunisia	8 soccer players from league 1 of the National senior league	CWI: 8 TWI: 8	19.6 ± 0.74 years	Simulated football match using the LIST	Ex: 10 min CWI, 10 °C Ex2: 10 min TWI, 28 °C	48 h	MVC; CMJ 20 m-sprint CK; MS
Brownstein	2019	United Kingdom	11 male semi-professional soccer players from level eight of the English football league	PCMcold: 11 CON: 11	22 ± 1 year	Football match	Ex: 3 h PCM(cold), 15 °C PAS: 3 h PCM, ambient temperature	48 h	CMJ; MVC
Clifford	2018	United Kingdom	11 elite male field players from the U-23 teams of the second-tier English league	PCMcold: 11 CON: 11	19 ± 1 year	Football match (≥60 min)	Ex: 3 h PCM(cold), 15 °C PAS: 3 h PCM, ambient temperature	36 h	CMJ; MVC MS
Cockburn	2013	United Kingdom	14 semi-professional	CHP: 7 CON: 7	24 ± 4 years	Eccentric-centripetal compound motion mode + LIST	Ex: CHP, milk PAS: Water	48 h	CMJ
Daab	2021	Tunisia	12 semi-professional Tunisian football players	IVO: 12 CON: 12	23 ± 1 year	Simulated football match using the LIST	Ex: IVO, three cycles of lower extremity vascular occlusion intervention (5 min of occlusion + 5 min of reperfusion, with a pressure of systolic pressure + 50 mmHg) PAS: Only the occlusion pressure was 20 mmHg (with no actual vascular occlusion effect)	48 h	CMJ; MVC 20-m sprint CK
Daab	2025	Tunisia	28 male soccer players from the second division of the National League	Hyp: 14 CON: 14	24.5 ± 3.6 years	Simulated football match using the LIST	Hyperoxic gas: Immediately and for the following 3 days, inhale a 99.5% hyperoxic gas mixture for 15 min/day PAS: Inhale a 21% normoxic gas mixture (e.g., indoor air) for 15 min	48 h	MVC; CMJ 20-m sprint CK; MS
Fullagar	2016	Germany	20 highly trained semi-professional male soccer players from the 5th and 6th tier leagues	SHS: 20 CON: 20	25.5 ± 4.6 years	Football match	SHS: Entering the bedroom before 23:45, dimming the lights, providing earplugs/eye masks, prohibiting the use of electronic devices 30 min before going to bed, turning off the lights and sleep at 00:00, and waking up at 07:30 the next day PAS: (NSHS)Freely use electronic devices. Lights will be turned off at 02:00 and woken up at 07:30 the next day (simulating the regular post-match schedule of players)	36,44 h	CMJ; CK
Goulart	2020	Brazil	10 professional female soccer players	RT: 10 CON: 10	25.1 ± 5.9 years	Football match	Ex: RT, after match 24 and 48 h PAS: No RT	48 h	CMJ 20-m sprint
Maior	2020	Brazil	20 professional football players from the Brazilian serie A (elite level)	INPT: 10 ACT: 10	21.0 ± 4.01 yeaear	Football match	Ex: INPT, 30 min of intermittent negative pressure therapy 24 h post-match Ex2: ACT, 30 min foam, core stability/flexibility training + low-intensity bicycle	24 h	CK
Mujika	2000	Spain	17 high-level male football players in national-level leagues	Cre: 8 CON: 9	20.3 ± 1.4 years	CMJT + RST + IET	Ex: Creatine, supply for 6 consecutive days, four times a day, 5 g each time (total dose 20 g per day) PAS: Maltodextrin, (consistent in appearance with creatine supplements)	24 h	CMJ
Nasser	2023	Tunis	12 semi-professional football player	CWI: 12 CON: 12	21.1 ± 2.2 years	Simulated football match using the LIST	Ex: 15 min CWI 11.3 °C ± 0.2 °C Ex2: PAS	48 h	CK; CMJ 20-m sprint
Rey	2012b	Spain	31 professional soccer players	ACT: 15 CON: 16	23.6 ± 3.4 years	Training + football match	Ex: ACT Ex2: PAS	24 h	MS
Rey	2012	Spain	31 professional soccer players	ACT: 15 CON: 16	23.6 ± 3.4 years	Training + football match	Ex: ACT Ex2: PAS	24 h	CMJ 20-m sprint
Rey	2019	Spain	18 professional soccer players	FR: 9 CON: 9	24.9 ± 4.42 years	Standard soccer training consisting of a 60 min program, including continuous dribbling/passing combination play, “Rondo” game, runs and high intensity positional 9 vs. 9 game	Ex: FR Ex2: PAS	24 h	CMJ
Shiravand	2024	Iran	16 male soccer players from Under-21 Iran’s premier league	atDCS: 16 CON: 16	20.9 ± 2.12 years	Football match stimulation	Ex: 20 min/time a-tDCS, (after match immediately, 24, 48 h) PAS: Sham tDCS	48 h	CMJ
Tessitore	2008	Italy	10 male futsal players from Italian futsal league and the European University Championship	ACT: 10 TWI: 10 Elec: 10 CON: 10	23 ± 2 years	Football match	Ex1: 20 min ACT Ex2: 20 min TWI, 30 °C Ex3: 20 min Electrostimulation Ex4: PAS	20 h	CMJ
Tseng	2024	China	12 elite athletes of Taiwan’s top women’s football league	FIR: 12 CON: 12	21.3 ± 1.15 years	Simulated football match using the LIST	Ex: 1 h/time FIR, was performed five times at 2, 25, 49, 73 and 97 h respectively PAS: Sham treatment	48 h	MVC; MS CK; CMJ
von stengel	2018	Germany	8 male football player from the fifth tier of the German football league	DO: 8 CON: 8	22 ± 3.3 years	90 min of specialized football training, including agility, sprinting, strength training and tactical confrontation	Ex: 15 min DO; twice/day (a total of four interventions) PAS: No recovery intervention	48 h	CK

ACT, active recovery; atDCS, transcranial direct current stimulation; CHO, Carbohydrate-electrolyte; CHP, Carbohydrate-protein; CK, creatine kinase; CMJ, countermovement jump height; CMJT, Counter-movement jump test; CON, passive recovery; Cre, Creatine; CrI, credible interval; Cryo, Cryo-compression; CWI, Cold-water immersion; DIC, deviance information criterion; DO, deep oscillation; Elec, Electrostimulation; FIR, Far-Infrared Radiation; FR, foam rolling; Hyp, Hyperoxic gas; ICWI, Intermittent cold-water immersion; IET, intermittent endurance test; INPT, intermittent negative pressure therapy; IVO, intermittent vascular occlusion; LIST, loughborough intermittent shuttle test; MCMC, markov chain monte carlo; MS, muscle soreness; MVC, maximal voluntary contraction; NSHS, no sleep hygiene strategy; PAS, passive recovery; PCMcold, Phase change material; RT, resistance training; RST, repeated sprint test; TWI, thermoneutral water immersion; RCT, randomized controlled trials; SHS, sleep hygiene strategy.

### Consistency analysis

Comparison of Deviance Information Criterion (DIC) values between consistency and inconsistency models indicated good agreement across all outcomes analyzed, including CMJ, CK, 20-m sprint, MVC, and MS (see Supplementary Table S6 of Supplementary Material S3).

### Effects of different interventions on CMJ

**FIGURE 2:**
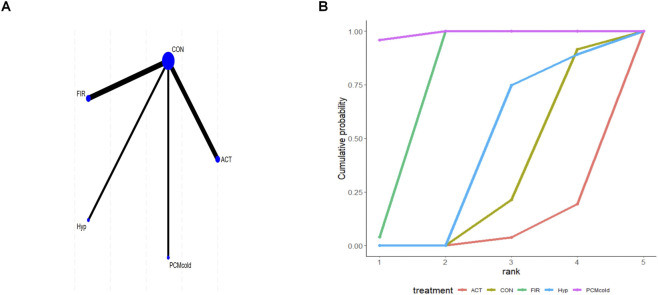
Analysis of countermovement jump height **(A)** Network plot; **(B)** Surface under the cumulative ranking curve.

Nineteen studies reported CMJ outcomes. Direct comparisons were made between the outcomes of all intervention types in the trials and those of control participants. As shown in the network plot, ACT, CWI, and the control group (Markus et al., 2021) formed a closed loop; therefore, local inconsistency testing was conducted (Figure 2A). No inconsistency was detected between direct, indirect, and network comparisons for CON vs. ACT, TWI vs. ACT, CWI vs. CON, or TWI vs. CWI (Supplementary Figure S3A of Supplementary Material S4). The mean differences (MD) represents the mean differences between two variables. For CMJ, higher values indicate greater muscle power or strength. According to the league table, compared with CON, ACT [MD = 2.04; 95% CrI (0.31, 3.79)] significantly improved CMJ (Supplementary Table S7A of Supplementary Material S5). Between interventions, FIR was superior to ACT, atDCS, CHP, Cre, Cryo, CWI, Elec, FR, and Hyp; IVO was superior to ACT, atDCS, CHP, Cryo, CWI, Elec, and FR; and PCMcold was superior to Elec. ACT, FIR, IVO, and PCMcold outperformed RT; FIR, IVO, and PCMcold were superior to SHS; and CWI, FIR, IVO, and PCMcold outperformed TWI. SUCRA rankings identified FIR (98.3%) as the most effective intervention, followed by IVO (91.9%), with RT (17.0%) as the least effective (Figure 2B; Supplementary Table S8 of Supplementary Material S6). Among interventions with statistically significant effects on CMJ, FIR has the highest SUCRA ranking (98.3%), supported by well-connected network structure. This suggests FIR may be a relatively more effective option for CMJ recovery, but conclusions should be tempered by differences in intervention protocols across studies.

### Effects of different interventions on CK

**FIGURE 3:**
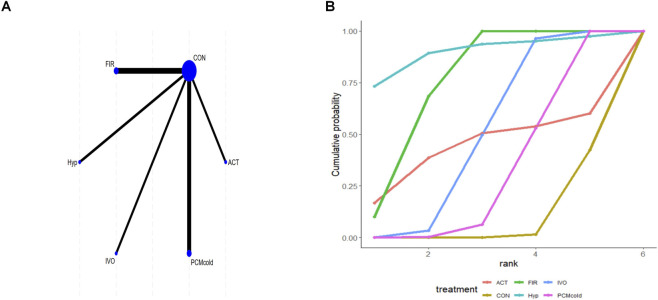
Analysis of creatine kinase **(A)** Network plot; **(B)** Surface under the cumulative ranking curve.

Twelve studies reported CK outcomes. Among the trials, the outcomes of all intervention types were directly compared with the outcomes of control participants. ACT, CWI, and CON formed a closed loop, allowing local inconsistency testing (Figure 3A). No inconsistency was observed for CWI vs. ACT (Supplementary Figure S3B of Supplementary Material S4). For CK, the smaller the absolute value represents the better effect of the intervention measures. Compared with CON, IVO [MD = −21.87; 95% CrI (−42.27, −1.42)] significantly reduced CK levels. CWI was superior to Hyp; CON, CWI, FIR, INPT was superior to IVO; INPT was superior to SHS; CWI, FIR, Hyp, INPT was outperformed TWI. SUCRA rankings identified INPT (91.0%) showed the most pronounced effect in reducing serum CK concentrations, followed by DO (88.2%), with Hyp (8.3%) as the least effective (Figure 3B; Supplementary Table S8 of Supplementary Material S6). Among CK-targeting interventions with significant effects, FIR has the highest SUCRA (98.3%, well-connected network), but conclusions are tempered by inter-study protocol differences.

### Effects of different interventions on 20-m sprint

**FIGURE 4:**
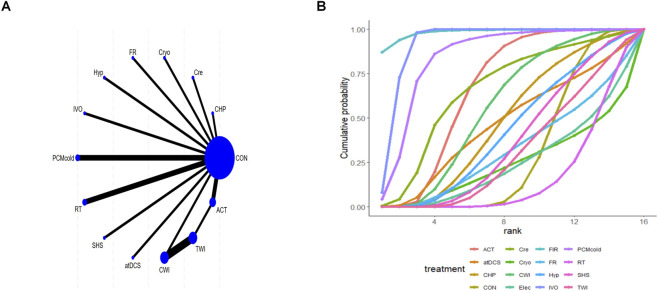
Analysis of 20-m sprint **(A)** Network plot; **(B)** Surface under the cumulative ranking curve.

Nine studies reported 20-m sprint outcomes. The outcomes of every intervention type participating in the trials were directly evaluated against those of control participants. However, no complete network was formed (Figure 4A). Based on the league table, no intervention significantly improved 20-m sprint performance (Supplementary Table S7C of Supplementary Material S5). SUCRA rankings placed IVO (84.5%) first, followed by CWI (51.2%), with TWI (23.9%) as the least effective (Figure 4B; Supplementary Table S8 of Supplementary Material S6). However, SUCRA rankings are purely descriptive in nature, as the sparsity or disconnectedness of the network, coupled with non-significant treatment effects, can compromise the robustness of the supporting evidence.

### Effects of different interventions on MVC

**FIGURE 5:**
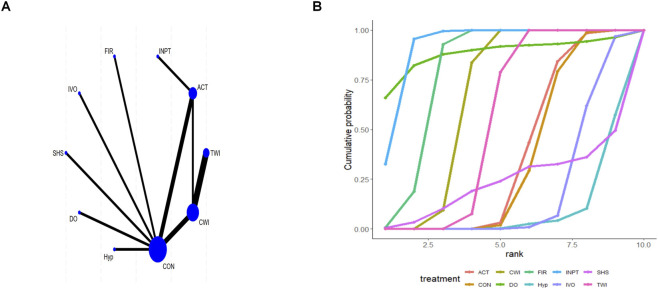
Analysis of maximal voluntary contraction **(A)** Network plot; **(B)** Surface under the cumulative ranking curve.

Six studies reported MVC, and direct comparisons were achieved among the outcomes of all intervention types in the trials and those of control participants. But no complete network structure was formed (Figure 5A). For MVC, the larger the absolute value, the more obvious the difference between the two variables. Relative to CON, FIR [MD = −38.43; 95% CrI (−52.63, −24.11)], IVO [MD = −15.82; 95% CrI (−19.91, −11.70)], and PCMcold [MD = −10.70; 95% CrI (−16.27, −5.12)] significantly improved MVC (Supplementary Table S7D of Supplementary Material S5). Additionally, FIR was superior to both IVO [MD = 22.61; 95% CrI (7.75, 37.38)] and PCMcold [MD = 27.72; 95% CrI (12.40, 42.96)]. SUCRA rankings indicated Hyp (89.8%) may be a relatively effective intervention, followed by FIR (75.7%), with CON (8.8%) as the least effective (Figure 5B; Supplementary Table S8 of Supplementary Material S6). However, SUCRA rankings are purely descriptive, as network disconnectedness and non-significant effects may weaken supporting evidence robustness.

### Effects of different interventions on MS

**FIGURE 6:**
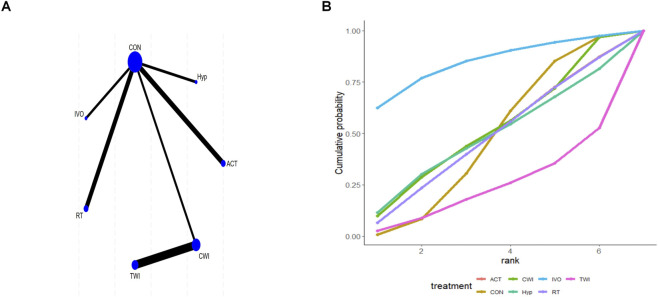
Analysis of muscle soreness **(A)** Network plot; **(B)** Surface under the cumulative ranking curve.

Five studies reported MS outcomes. In these trials, the outcomes of all intervention types were directly compared with the outcomes of control group. All comparisons involved CON, with no direct comparisons between interventions (Figure 6A). For MS, the smaller the absolute value represents the better effect of the intervention measures. Relative to CON, FIR [MD = 16.82; 95% CrI (12.33, 21.31)] and PCMcold [MD = 32.92; 95% CrI (15.41, 50.54)] significantly reduced MS. FIR was superior to ACT [MD = 17.07; 95% CrI (12.57, 21.57)], while PCMcold was superior to ACT [MD = 33.18; 95% CrI (15.66, 50.81)] and Hyp [MD = 32.65; 95% CrI (15.12, 50.30)] (Supplementary Table S7E of Supplementary Material S5). SUCRA rankings identified PCMcold (98.9%) as the most effective intervention, followed by FIR (76.0%), with ACT (5.8%) as the least effective (Figure 6B; Supplementary Table S8 of Supplementary Material S6).

### Publication bias

Funnel plot analyses indicated a low risk of publication bias for CMJ, CK, 20-m sprint, MVC, and MS (Supplementary Figure S4 of Supplementary Material S8).

## Discussion

This study evaluated the effects of 17 recovery interventions on soccer-induced fatigue in elite players, synthesizing evidence from 23 randomized controlled trials involving 388 participants. The findings show that FIR demonstrated the most favorable effect on CMJ recovery; INPT was likely the most effective intervention for reducing CK; no intervention produced significant benefits for 20-m sprint performance (despite IVO ranking first, the result lacked reliability); Hyp was potentially optimal for improving MVC; and PCMcold showed the greatest efficacy for reducing muscle soreness. These results provide evidence-based guidance for personalized recovery strategy selection while underscoring key limitations within the existing literature.

Post-match CMJ decline is primarily caused by skeletal muscle microdamage, inflammation, metabolic by-product accumulation, and neuromuscular inhibition (Nédélec et al., 2012). The superior performance of FIR likely reflects the synergy of its thermal and non-thermal mechanisms: thermal effects induce microvascular dilation to enhance oxygen and nutrient delivery (Lin et al., 2007), while non-thermal effects modulate membrane potential and mitochondrial metabolism to accelerate metabolic waste clearance (Shui et al., 2015; Vatansever and Hamblin, 2012). FIR also promotes calcium-dependent nitric oxide release, attenuating inflammation and improving neuromuscular transmission (Leung et al., 2009). These mechanisms collectively facilitate lower-limb power recovery. Consistent with Loturco et al. (Loturco et al., 2016), the present NMA extends prior evidence by confirming FIR’s superiority relative to 16 other interventions through combined direct and indirect comparisons.

CK is a well-established biomarker of myofibrillar membrane disruption, and elevated serum concentrations indicate compromised muscle membrane integrity (Greenham et al., 2018). INPT’s robust CK-lowering effect may arise from its ability to decrease leukocyte-mediated structural damage to muscle fibers, stabilize cell membranes (thereby reducing cytosolic CK release into the bloodstream), and enhance venous return to clear extracellular CK and metabolic by-products (Magalhães et al., 2009; Baird et al., 2012; Pfirrmann et al., 2016). INPT outperformed conventional modalities such as CWI and FIR, aligning with findings from Wiecha et al. (2021), who emphasized negative pressure therapy’s dual benefits for microcirculation enhancement and inflammation suppression. Its portability and non-invasiveness further underscore its practical value in elite sports settings with congested match schedules.

The absence of significant effects on 20-m sprint performance is likely attributable to three main limitations (Stølen et al., 2005): Incomplete network structure: Most studies compared a single intervention with CON, yielding sparse indirect comparisons and heterogeneous control conditions (Nédélec et al., 2012). Insufficient statistical power: Small samples and high inter-individual variability in sprint performance hindered detection of small but meaningful effects (Bush et al., 2015). Masking by natural recovery processes: Most included studies assessed sprint performance at 48 h post-match, when neuromuscular fatigue naturally recovers by 70%–80% (Nédélec et al., 2012), reducing the observable effect of interventions. Thus, although IVO ranked highest in SUCRA, the finding is unreliable due to incomplete network architecture. Practically, CWI remains a pragmatic option because of its accessibility, low cost, and strong athlete acceptance.

MVC decline after soccer results from both central (reduced motor cortex activation) and peripheral mechanisms (structural damage, inflammation) (Daab et al., 2021). The potential benefits of Hyp are linked to oxygen homeostasis regulation and enhanced muscle repair. Moderate hypoxic stress activates HIF-1α signaling, promoting angiogenesis, satellite cell proliferation, myoblast differentiation, and myofiber regeneration (Warren et al., 1999), while simultaneously moderating inflammatory responses (e.g., limiting IL-6 overproduction). However, conclusions remain tentative due to limited sample sizes and incomplete networks (e.g., no direct comparisons between Hyp and FIR). Personalized adjustments based on hyperoxic tolerance are recommended, and future multi-arm RCTs are necessary to validate its clinical utility.

Muscle soreness is largely driven by secondary muscle damage mediated by immune activation. Following microdamage, neutrophils infiltrate affected tissues and release reactive oxygen species (ROS), exacerbating soreness (Schaser et al., 2007; Toumi HaB and Best, 2003). PCMcold (∼15 °C) maintains localized cooling for up to 3 h, reducing inflammatory cell adhesion and infiltration while limiting ROS production, thereby mitigating secondary damage (Paulsen et al., 2010). The 48-h measurement time in this study aligns with the peak inflammatory response (24–96 h), reinforcing the observed analgesic effect. The device’s portability and comfort also promote adherence, with perceptual benefits potentially contributing to symptom relief (Bibić et al., 2025). These findings align with prior research (Kwiecien et al., 2018) and further establish PCMcold’s superiority over FIR and Hyp through NMA.

### Clinical implications

Based on the synthesized evidence, the following clinical recommendations for post-match recovery in elite soccer players are proposed (Stølen et al., 2005): Neuromuscular power recovery (e.g., within 48 h post-match): Prioritize far-infrared therapy (FIR), as the synergistic effects of its thermal and non-thermal mechanisms facilitate rapid improvements in CMJ performance. Combined with the minimal clinically important difference of CMJ set at 3.9 cm for athletes (Pojskić et al., 2025), the absolute MD between FIR and IVO was 4.84 cm (exceeding MD), suggesting that FIR confers clinically meaningful advantages in mitigating CMJ decline. In contrast, the MD difference between IVO and PCMcold was less than MD, indicating their clinical effects are comparable in practical application (Nédélec et al., 2012). Reduction of muscle damage biomarkers (e.g., after high-intensity derby matches): Recommend intermittent negative pressure therapy (INPT) for maximal CK-lowering efficacy, or cold-water immersion (CWI) as a cost-effective and accessible alternative to reduce serum CK concentrations (Bush et al., 2015). Subjective muscle soreness relief: Prefer portable cold compression therapy (PCMcold), particularly for athletes who are intolerant to CWI (Markus et al., 2021). Muscle strength recovery: FIR is a reliable option based on significant pairwise comparisons, while hyperoxic gas (Hyp) may be considered for athletes with sufficient hyperoxic tolerance (Querido et al., 2022). 20-m sprint recovery: No single intervention demonstrated clear superiority. Basic recovery measures, including adequate sleep and nutritional support, are recommended; complex or resource-intensive modalities should not be over-relied upon. It should be emphasized that combined recovery strategies (e.g., PCMcold plus protein supplementation) may offer additive benefits. However, current evidence remains insufficient to provide definitive guidance on multi-component protocols, highlighting an important direction for future research. Finally, the interventions recommended in this study are mainly suitable for delayed recovery (∼48 h post-match); optimal strategies for acute recovery (∼30 min post-match) require further validation with more studies.

### Limitations

This study has several key limitations: First, methodological constraints of RCT in elite team sport settings: High-density competitive schedules often limit the feasibility of intervention implementation, resulting in small sample sizes. Variability in individual training loads and adherence further complicates standardization. Notably, studies examining fatigue from official matches are scarce; most rely on laboratory-based simulations that differ from real matches in tactical intensity and metabolic load, potentially reducing ecological validity. Second, potential bias from data standardization: Measurement time points for MVC varied slightly across studies (e.g., 44–45 h post-match), necessitating approximation to 48 h for pooled analysis. While this approach is common in NMA, it may introduce minor systematic errors. Nevertheless, effect size distributions suggest minimal impact on overall conclusions. Last, scope of intervention types: Due to the requirement for clear, comparable nodes in NMA, combined recovery strategies (e.g., CWI plus nutritional supplementation) were excluded. Heterogeneity in intervention components and implementation sequences, coupled with inconsistent reporting, precludes meaningful network construction. Therefore, conclusions are primarily applicable to single-intervention scenarios, and caution is warranted when generalizing to multi-modal recovery protocols.

## Conclusion

This systematic review and network meta-analysis synthesized evidence from 23 studies to comprehensively compare recovery strategies, including ACT, CWI, Cryo, TWI, PCMcold, FIR, and IVO, etc., on physiological, neuromuscular, and subjective outcomes in elite soccer players. Key findings include: FIR was the most effective intervention for improving CMJ performance. INPT demonstrated the greatest efficacy in reducing CK, a biochemical marker of muscle damage. No intervention produced significant improvements in 20-m sprint performance. Hyp appeared potentially optimal for enhancing MVC. PCMcold was most effective for alleviating muscle soreness.

## Data Availability

The original contributions presented in the study are included in the article/Supplementary Material, further inquiries can be directed to the corresponding author.
