# Supplementary material for: Efficacy of post-exercise recovery strategies for elite soccer players: a network meta-analysis
Source: Front Physiol. 2026 Jan 21;17:1760392. doi: 10.3389/fphys.2026.1760392 (PMC12867776; doi:10.3389/fphys.2026.1760392)
Supplement: Supplementary file 1 [file DataSheet1.docx]

***Supplementary materials***

**Supplementary materials 1. Search strategy**

**Search strategy and history (Until October, 2025)(1)**

**Search strategy Table 1.** Search strategy in Pubmed.

(1) OR (soccer[MeSH Terms])) OR ((football players[Title/Abstract])(1) OR (soccer players[Title/Abstract]))) AND (match OR game OR competition OR post-match OR post-game OR post competition)(1)) AND (((recovery of function[MeSH Terms]) OR (massage[MeSH Terms])) OR (((((((((((((((((((((recovery of function[Title/Abstract]) OR (recover*[Title/Abstract])) OR (cold-water immersion[Title/Abstract])) OR (active recovery[Title/Abstract])) OR (passive recovery[Title/Abstract])) OR (stretch*[Title/Abstract])) OR (compression garments[Title/Abstract])) OR (whole-body vibration[Title/Abstract])) OR (electrostimulation[Title/Abstract])) OR (foam*[Title/Abstract])) OR (contrast baths[Title/Abstract])) OR (chamber[Title/Abstract])) OR (cool*[Title/Abstract])) OR (warm*[Title/Abstract])) OR (blood flow restriction[Title/Abstract])) OR (nutrition[Title/Abstract])) OR (supplement*[Title/Abstract])) OR (sleep[Title/Abstract])) OR (neuromuscular recovery[Title/Abstract])) OR (cryotherapy[Title/Abstract])) OR (cold therapy*[Title/Abstract])))) AND (((((((((((((((((((((performance[Title/Abstract]) OR (muscle soreness[Title/Abstract])) OR (muscle damage[Title/Abstract])) OR (delayed onset muscle soreness(DOMS[Title/Abstract]))) OR (heart rate[Title/Abstract])) OR (tendon damage[Title/Abstract])) OR (counter movement jump(CMJ[Title/Abstract]))) OR (sprint[Title/Abstract])) OR (agility[Title/Abstract])) OR (biochemical markers[Title/Abstract])) OR (creatine kinase(CK[Title/Abstract]))) OR (cortisol[Title/Abstract])) OR (testosterone[Title/Abstract])) OR (interleukin-6(IL-6[Title/Abstract]))) OR (perceived fatigue[Title/Abstract])) OR (perceived*[Title/Abstract])) OR (C-reactive protein(CRP[Title/Abstract]))) OR (perception[Title/Abstract])) OR (wellness[Title/Abstract])) OR (sleep[Title/Abstract])) OR (total quality recovery[Title/Abstract]))) AND (((randomized controlled trial[Publication Type]) OR (randomized[Title/Abstract])) OR (placebo[Title/Abstract]))

There were 243 records identified through PubMed database searching.

**Search strategy Table 2.** Search strategy in Web of science.

| **#** | **Search Query** | **Database** | **Results** |
| --- | --- | --- | --- |
| 1 | (((TS=(football)) OR TS=(soccer)) OR TS=(football players)) OR TS=(soccer players) | Web of Science Core Collection | 58613 |
| 2 | (((((((((((((((((((((TS=(recovery of function)) OR TS=(massage)) OR TS=(recover*)) OR TS=(cold-water immersion)) OR TS=(active recovery)) OR TS=(passive recovery)) OR TS=(stretch*)) OR TS=(compression garments )) OR TS=(whole-body vibration)) OR TS=(electrostimulation)) OR TS=(foam*)) OR TS=(contrast baths)) OR TS=(contrast baths)) OR TS=(contrast baths)) OR TS=(warm*)) OR TS=(blood flow restriction)) OR TS=(nutrition)) OR TS=(supplement*)) OR TS=(sleep)) OR TS=(neuromuscular recovery)) OR TS=(cryotherapy)) OR TS=(cold therapy*) | Web of Science Core Collection | 3625624 |
| 3 | ((((((((((((((((((((TS=(performance )) OR TS=(muscle soreness)) OR TS=(muscle damage)) OR TS=(delayed onset muscle soreness(DOMS))) OR TS=(heart rate)) OR TS=(tendon damage)) OR TS=(counter movement jump(CMJ))) OR TS=(sprint)) OR TS=(agility)) OR TS=(biochemical markers)) OR TS=(creatine kinase(CK) )) OR TS=(cortisol )) OR TS=(testosterone )) OR TS=(interleukin-6(IL-6))) OR TS=(perceived fatigue)) OR TS=(perceived*)) OR TS=(C-reactive protein(CRP))) OR TS=(perception)) OR TS=(wellness)) OR TS=(sleep)) OR TS=(total quality recovery) | Web of Science Core Collection | 7965754 |
| 4 | (((((TS=(match)) OR TS=(game )) OR TS=(competition )) OR TS=(post-match)) OR TS=( post-game )) OR TS=(post competition) | Web of Science Core Collection | 1678203 |
| 5 | (((((TS=(randomized controlled trial)) OR TS=(randomized)) OR TS=(placebo)) OR TS=(random)) OR TS=(placebo)) OR TS=(double-blind) | Web of Science Core Collection | 2349471 |
| 6 | #1 AND #2 AND #3 AND #4 AND #5 | Web of Science Core Collection | 333 |

There were 333 records identified through Web of science database searching.

**Search strategy Table 3.** Search strategy in Embase.

| **No.** | **Query** | **Results** | **Date** |
| --- | --- | --- | --- |
| #69 | #61 AND #68 | 98 | 13-Nov-25 |
| #68 | #62 OR #63 OR #64 OR #65 OR #66 OR #67 | 281393 | 13-Nov-25 |
| #67 | 'post competition':ab,ti | 212 | 13-Nov-25 |
| #66 | 'post-game':ab,ti | 236 | 13-Nov-25 |
| #65 | 'post-match':ab,ti | 547 | 13-Nov-25 |
| #64 | 'competition':ab,ti | 126671 | 13-Nov-25 |
| #63 | 'game':ab,ti | 48702 | 13-Nov-25 |
| #62 | 'match':ab,ti | 110917 | 13-Nov-25 |
| #61 | #56 AND #60 | 336 | 13-Nov-25 |
| #60 | #57 OR #58 OR #59 | 1079848 | 13-Nov-25 |
| #59 | 'double-blind':ab,ti | 299491 | 13-Nov-25 |
| #58 | 'placebo':ab,ti | 470258 | 13-Nov-25 |
| #57 | 'random':ab,ti | 534689 | 13-Nov-25 |
| #56 | #7 AND #54 AND #55 | 3013 | 13-Nov-25 |
| #55 | #10 OR #32 | 3064267 | 13-Nov-25 |
| #54 | #33 OR #34 OR #35 OR #36 OR #37 OR #38 OR #39 OR #40 OR #41 OR #42 OR #43 OR #44 OR #45 OR #46 OR #47 OR #48 OR #49 OR #50 OR #51 OR #52 OR #53 | 3511066 | 13-Nov-25 |
| #53 | 'total quality recovery':ab,ti | 60 | 13-Nov-25 |
| #52 | 'sleep':ab,ti | 401207 | 13-Nov-25 |
| #51 | 'wellness':ab,ti | 25861 | 13-Nov-25 |
| #50 | 'perception':ab,ti | 312582 | 13-Nov-25 |
| #49 | 'c-reactive protein(crp)':ab,ti | 64623 | 13-Nov-25 |
| #48 | 'perceived*':ab,ti | 396206 | 13-Nov-25 |
| #47 | 'perceived fatigue':ab,ti | 980 | 13-Nov-25 |
| #46 | 'interleukin-6(il-6)':ab,ti | 48727 | 13-Nov-25 |
| #45 | 'testosterone':ab,ti | 129067 | 13-Nov-25 |
| #44 | 'cortisol':ab,ti | 102897 | 13-Nov-25 |
| #43 | 'creatine kinase(ck)':ab,ti | 11889 | 13-Nov-25 |
| #42 | 'biochemical markers':ab,ti | 24555 | 13-Nov-25 |
| #41 | 'agility':ab,ti | 6444 | 13-Nov-25 |
| #40 | 'sprint':ab,ti | 12427 | 13-Nov-25 |
| #39 | 'counter movement jump(cmj)':ab,ti | 274 | 13-Nov-25 |
| #38 | 'tendon damage':ab,ti | 317 | 13-Nov-25 |
| #37 | 'heart rate':ab,ti | 285615 | 13-Nov-25 |
| #36 | 'delayed onset muscle soreness(doms)':ab,ti | 785 | 13-Nov-25 |
| #35 | 'muscle damage':ab,ti | 8720 | 13-Nov-25 |
| #34 | 'muscle soreness':ab,ti | 3506 | 13-Nov-25 |
| #33 | 'performance':ab,ti | 1945292 | 13-Nov-25 |
| #32 | #10 OR #11 OR #12 OR #13 OR #14 OR #15 OR #16 OR #17 OR #18 OR #19 OR #20 OR #21 OR #22 OR #23 OR #24 OR #25 OR #26 OR #27 OR #28 OR #29 OR #30 OR #31 | 3064267 | 13-Nov-25 |
| #31 | 'cold therapy*':ab,ti | 454 | 13-Nov-25 |
| #30 | 'cryotherapy':ab,ti | 14411 | 13-Nov-25 |
| #29 | 'neuromuscular recovery':ab,ti | 419 | 13-Nov-25 |
| #28 | 'sleep':ab,ti | 401207 | 13-Nov-25 |
| #27 | 'supplement*':ab,ti | 645303 | 13-Nov-25 |
| #26 | 'nutrition':ab,ti | 326271 | 13-Nov-25 |
| #25 | 'blood flow restriction':ab,ti | 1990 | 13-Nov-25 |
| #24 | 'warm*':ab,ti | 131241 | 13-Nov-25 |
| #23 | 'cool*':ab,ti | 95970 | 13-Nov-25 |
| #22 | 'chamber':ab,ti | 164835 | 13-Nov-25 |
| #21 | 'contrast baths':ab,ti | 47 | 13-Nov-25 |
| #20 | 'foam*':ab,ti | 51901 | 13-Nov-25 |
| #19 | 'electrostimulation':ab,ti | 5256 | 13-Nov-25 |
| #18 | 'whole-body vibration':ab,ti | 3562 | 13-Nov-25 |
| #17 | 'compression garments':ab,ti | 884 | 13-Nov-25 |
| #16 | 'stretch*':ab,ti | 113032 | 13-Nov-25 |
| #15 | 'passive recovery':ab,ti | 1034 | 13-Nov-25 |
| #14 | 'active recovery':ab,ti | 1136 | 13-Nov-25 |
| #13 | 'cold-water immersion*':ab,ti | 1209 | 13-Nov-25 |
| #12 | 'recover*':ab,ti | 1226715 | 13-Nov-25 |
| #11 | 'recovery of function':ab,ti | 3443 | 13-Nov-25 |
| #10 | #8 OR #9 | 87203 | 13-Nov-25 |
| #9 | 'massage'/exp | 23560 | 13-Nov-25 |
| #8 | 'convalescence'/exp | 63796 | 13-Nov-25 |
| #7 | #3 OR #6 | 28568 | 13-Nov-25 |
| #6 | #4 OR #5 | 13262 | 13-Nov-25 |
| #5 | 'soccer players':ab,ti | 7836 | 13-Nov-25 |
| #4 | 'football players':ab,ti | 5836 | 13-Nov-25 |
| #3 | #1 OR #2 | 22854 | 13-Nov-25 |
| #2 | 'soccer'/exp | 9729 | 13-Nov-25 |
| #1 | 'football'/exp | 14935 | 13-Nov-25 |

There were 98 records identified through Embase database searching.

**Search strategy Table 4.** Search strategy in Cochrane Library.

ID Search Hits

#1 MeSH descriptor: [Soccer] explode all trees 1221

#2 MeSH descriptor: [Soccer] explode all trees 1221

#3 football players 1215

#4 soccer players 1921

#5 match 7048

#6 game 7292

#7 competition 2247

#8 post-match 54

#9 post-game 27

#10 post-game 27

#11 #1 or #2 or #3 or #4 2903

#12 #5 or #6 or #7 or #8 or #9 or #10 16187

#13 MeSH descriptor: [Recovery of Function] explode all trees 7501

#14 MeSH descriptor: [Massage] explode all trees 1777

#15 recovery of function 27010

#16 recover* 105132

#17 cold-water immersion 525

#18 active recovery 7627

#19 passive recovery 1828

#20 stretch* 14245

#21 compression garments 354

#22 whole-body vibration 1771

#23 electrostimulation 2770

#24 foam* 4170

#25 contrast baths 56

#26 chamber 8123

#27 cool* 8417

#28 warm* 15678

#29 blood flow restriction 3197

#30 nutrition 72468

#31 supplement* 113100

#32 sleep 64539

#33 neuromuscular 17476

#34 neuromuscular recovery 3586

#35 cryotherapy 3331

#36 cold therapy* 6278

#37 #13 or #14 or #15 or #16 or #17 or #18 or #19 or #20 or #21 or #22 or #23 or #24 or #25 or #26 or #27 or #28 or #29 or #30 or #31 or #32 or #33 or #34 or #35 or #36 377245

#38 performance 150712

#39 muscle soreness 2257

#40 muscle damage 4912

#41 delayed onset muscle soreness(DOMS) 557

#42 heart rate 113090

#43 tendon damage 364

#44 counter movement jump(CMJ) 151

#45 sprint 3997

#46 agility 2013

#47 biochemical markers 5432

#48 creatine kinase(CK) 2083

#49 cortisol 15199

#50 testosterone 9890

#51 interleukin-6(IL-6) 23761

#52 perceived fatigue 3777

#53 perceived* 44593

#54 C-reactive protein(CRP) 13392

#55 perception 38540

#56 wellness 3491

#57 sleep 64539

#58 total quality recovery 7670

#59 #38 or #39 or #40 or #41 or #42 or #43 or #44 or #45 or #46 or #47 or #48 or #49 or #50 or #51 or #52 or #53 or #54 or #55 or #56 or #57 or #58 421478

#60 #11 AND #12 AND #37 AND #59 294

There were 284 records identified through Cochrane Library database searching.

**Search strategy Table 5.** Search strategy in Scopus.

( ( TITLE-ABS-KEY ( football ) OR TITLE-ABS-KEY ( soccer ) OR TITLE-ABS-KEY ( football players ) OR TITLE-ABS-KEY ( football players ) ) ) AND ( ( TITLE-ABS-KEY ( recovery of function ) OR TITLE-ABS-KEY ( massage ) OR TITLE-ABS-KEY ( recover* ) OR TITLE-ABS-KEY ( cold-water immersion ) OR TITLE-ABS-KEY ( ACTIVE recovery ) OR TITLE-ABS-KEY ( passive recovery ) OR TITLE-ABS-KEY ( stretch* ) OR TITLE-ABS-KEY ( compression garments ) OR TITLE-ABS-KEY ( compression garments ) OR TITLE-ABS-KEY ( electrostimulation ) OR TITLE-ABS-KEY ( foam* ) OR TITLE-ABS-KEY ( contrast baths ) OR TITLE-ABS-KEY ( chamber ) OR TITLE-ABS-KEY ( cool* ) OR TITLE-ABS-KEY ( warm* ) OR TITLE-ABS-KEY ( blood flow restriction ) OR TITLE-ABS-KEY ( nutrition ) OR TITLE-ABS-KEY ( supplement* ) OR TITLE-ABS-KEY ( sleep ) OR TITLE-ABS-KEY ( neuromuscular recovery ) OR TITLE-ABS-KEY ( cryotherapy ) OR TITLE-ABS-KEY ( cold therapy* ) ) ) AND ( ( TITLE-ABS-KEY ( performance ) OR TITLE-ABS-KEY ( muscle soreness ) OR TITLE-ABS-KEY ( muscle damage ) OR TITLE-ABS-KEY ( delayed onset muscle soreness ) OR TITLE-ABS-KEY ( heart rate ) OR TITLE-ABS-KEY ( tendon damage ) OR TITLE-ABS-KEY ( counter movement jump ) OR TITLE-ABS-KEY ( sprint ) OR TITLE-ABS-KEY ( agility ) OR TITLE-ABS-KEY ( biochemical markers ) OR TITLE-ABS-KEY ( creatine kinase ) OR TITLE-ABS-KEY ( cortisol ) OR TITLE-ABS-KEY ( testosterone ) OR TITLE-ABS-KEY ( interleukin-6 ) OR TITLE-ABS-KEY ( perceived fatigue ) OR TITLE-ABS-KEY ( perceived* ) OR TITLE-ABS-KEY ( C-reactive protein ) OR TITLE-ABS-KEY ( perception ) OR TITLE-ABS-KEY ( wellness ) OR TITLE-ABS-KEY ( sleep ) OR TITLE-ABS-KEY ( total QUALITY recovery ) ) ) AND ( ( TITLE-ABS-KEY ( match ) OR TITLE-ABS-KEY ( game ) OR TITLE-ABS-KEY ( competition ) OR TITLE-ABS-KEY ( post-match ) OR TITLE-ABS-KEY ( post-game ) OR TITLE-ABS-KEY ( post competition ) ) ) AND ( ( TITLE-ABS-KEY ( randomized controlled trial ) OR TITLE-ABS-KEY ( randomized ) OR TITLE-ABS-KEY ( placebo ) OR TITLE-ABS-KEY ( random ) OR TITLE-ABS-KEY ( double-blind ) ) )

There were 324 records identified through Scopus database searching.

**Supplementary materials 2.** **Risk of bias**


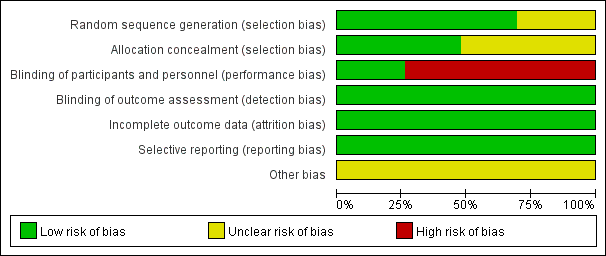


**Supplementary Fig. 1.** The overall risk of bias for all involved trails.


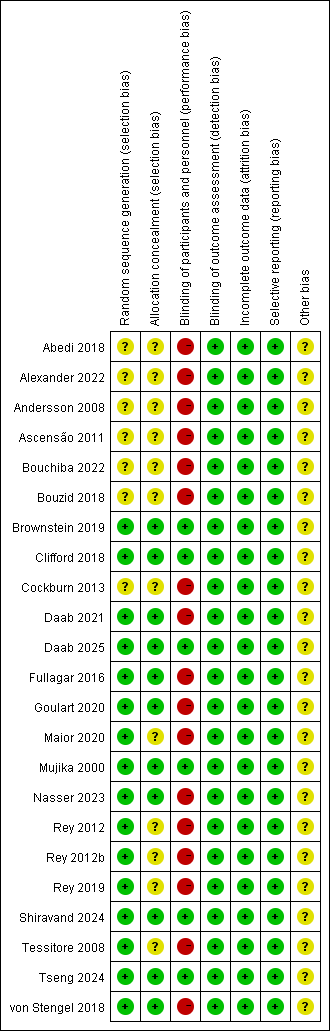


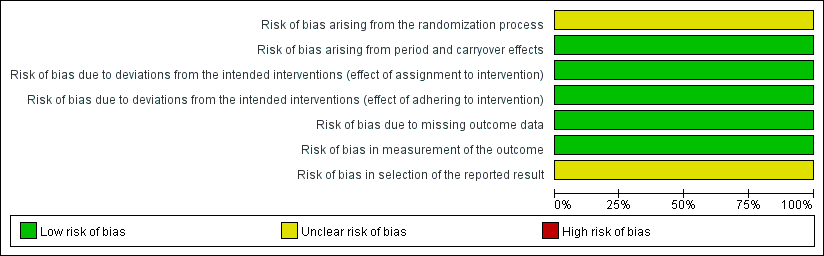
**Supplementary Fig. 2.** The overall risk of bias for cross-over trails.


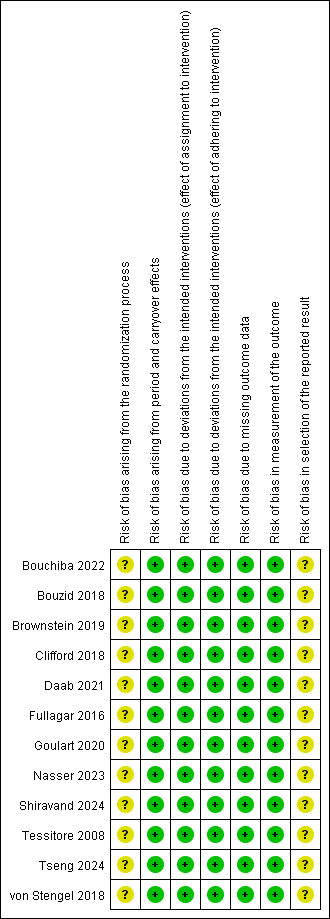


**Quality assessment of included studies**

For random sequence generation and allocation concealment, six studies were rated as “unclear” due to insufficient methodological details. In terms of blinding, six studies were judged as “low risk,” whereas most were rated as “high risk.” This was largely attributable to the inherent nature of the interventions—participants could easily identify their assigned groups based on sensory or procedural cues, and informed consent requirements limited the feasibility of blinding, thereby introducing performance bias. Regarding incomplete outcome data, all studies reported baseline and final sample sizes, with no missing outcome data. A few studies documented minor participant dropouts, but all provided clear explanations. For blinding of participants and personnel, several studies attempted to minimize performance bias by using strategies such as standardized intervention carriers (similar appearance or taste), informational guidance to reduce perceived differences between groups, or separating intervention administrators from outcome assessors. Detailed risk-of-bias evaluations are presented in Supplementary Fig 1 and 2 of Supplementary materials 2.

**Supplementary Fig. 2.** The risk of bias for each trial.

**Supplementary material 3. The comparison of the deviance information criterion (DIC) in consistency and inconsistency models.**

| **Outcomes** | **Consistency test** | **Inconsistency test** | **I2(%)** |
| --- | --- | --- | --- |
| CMJ | 92.59 | 92.50 | 27 |
| CK | 52.78 | 52.80 | 25 |
| 20m-sprint | 40.15 | 40.18 | 6 |
| MVC | 26.57 | 26.64 | 11 |
| MS | 23.77 | 23.81 | 20 |

**Supplementary Table. 6.** CMJ: Countermovement jump height; CK: Creatine kinase; MVC: Maximal voluntary contraction; MS: Muscle soreness

**Supplementary material 4. Node-splitting analysis**


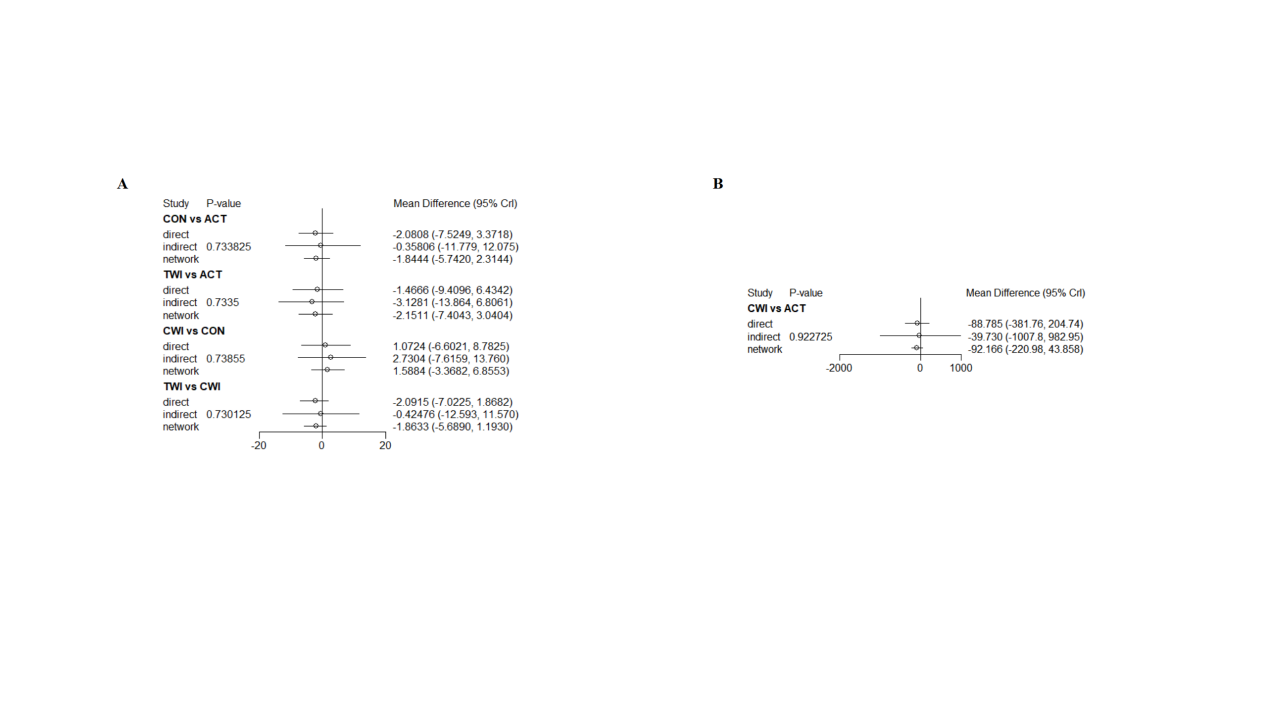


**Supplementary Fig. 3.** Node-splitting analysis in (A) Countermovement jump height(CMJ), (B) Creatine kinase(CK). For those P values exceeding 0.05, consistency models were adopted for the network meta-analyses that followed. ACT:Active recovery; CON:Passive recovery; CWI:Cold-water immersion; TWI:Thermoneutral water immersion;

**Supplementary material 5. Comparative effectiveness results of various types of exercise in primary outcomes**

A: CMJ League table

| MD 95%CRI | | | | | | | | | | | | | | | |
| --- | --- | --- | --- | --- | --- | --- | --- | --- | --- | --- | --- | --- | --- | --- | --- |
| ACT |  |  |  |  |  |  |  |  |  |  |  |  |  |  |  |
| 1.1 (-3.44, 5.65) | atDCS |  |  |  |  |  |  |  |  |  |  |  |  |  |  |
| 1.13 (-1.62, 3.9) | 0.04 (-4.71, 4.76) | CHP |  |  |  |  |  |  |  |  |  |  |  |  |  |
| 2.04 (0.31, 3.79)* | 0.93 (-3.26, 5.14) | 0.9 (-1.24, 3.06) | CON |  |  |  |  |  |  |  |  |  |  |  |  |
| -0.55 (-5.17, 4.06) | -1.66 (-7.65, 4.34) | -1.69 (-6.44, 3.08) | -2.6 (-6.83, 1.66) | Cre |  |  |  |  |  |  |  |  |  |  |  |
| 2.93 (-2.06, 7.91) | 1.83 (-4.45, 8.09) | 1.8 (-3.35, 6.95) | 0.89 (-3.78, 5.57) | 3.49 (-2.86, 9.8) | Cryo |  |  |  |  |  |  |  |  |  |  |
| 0.68 (-1.86, 3.2) | -0.43 (-5.24, 4.44) | -0.45 (-3.65, 2.75) | -1.36 (-3.72, 1.02) | 1.24 (-3.64, 6.12) | -2.25 (-7.49, 3) | CWI |  |  |  |  |  |  |  |  |  |
| 2.65 (-1.19, 6.48) | 1.54 (-3.86, 6.95) | 1.52 (-2.53, 5.54) | 0.61 (-2.81, 4.03) | 3.21 (-2.24, 8.66) | -0.28 (-6.08, 5.51) | 1.97 (-2.21, 6.14) | Elec |  |  |  |  |  |  |  |  |
| -9.26 (-16.99, -1.57)* | -10.36 (-18.98, -1.78)* | -10.39 (-18.22, -2.59)* | -11.3 (-18.8, -3.81)* | -8.71 (-17.33, -0.07)* | -12.19 (-21.04, -3.35)* | -9.93 (-17.83, -2.09)* | -11.9 (-20.17, -3.65)* | FIR |  |  |  |  |  |  |  |
| 2.13 (-1.92, 6.21) | 1.03 (-4.53, 6.61) | 1 (-3.25, 5.27) | 0.09 (-3.56, 3.79) | 2.69 (-2.91, 8.31) | -0.8 (-6.73, 5.15) | 1.45 (-2.9, 5.83) | -0.52 (-5.49, 4.5) | 11.39 (3.05, 19.79)* | FR |  |  |  |  |  |  |
| 1.44 (-1.54, 4.42) | 0.34 (-4.49, 5.16) | 0.3 (-2.92, 3.53) | -0.61 (-3.01, 1.82) | 1.99 (-2.9, 6.88) | -1.49 (-6.73, 3.76) | 0.76 (-2.62, 4.15) | -1.22 (-5.4, 3) | 10.69 (2.81, 18.59)* | -0.7 (-5.08, 3.69) | Hyp |  |  |  |  |  |
| -4.42 (-6.73, -2.11)* | -5.52 (-9.98, -1.07)* | -5.55 (-8.19, -2.93)* | -6.46 (-7.96, -4.96)* | -3.86 (-8.38, 0.63) | -7.35 (-12.24, -2.45)* | -5.1 (-7.92, -2.28)* | -7.07 (-10.81, -3.33)* | 4.84 (-2.82, 12.49) | -6.56 (-10.53, -2.59)* | -5.86 (-8.71, -3.03)* | IVO |  |  |  |  |
| -2.96 (-7.49, 1.55) | -4.06 (-9.97, 1.88) | -4.09 (-8.79, 0.62) | -5 (-9.18, -0.81)* | -2.4 (-8.34, 3.56) | -5.89 (-12.17, 0.36) | -3.64 (-8.42, 1.16) | -5.6 (-11.02, -0.22)* | 6.3 (-2.22, 14.89) | -5.08 (-10.63, 0.45) | -4.4 (-9.21, 0.43) | 1.46 (-2.98, 5.9) | PCMcold |  |  |  |
| 2.81 (0.64, 4.97)* | 1.7 (-2.69, 6.09) | 1.67 (-0.84, 4.18) | 0.77 (-0.53, 2.06) | 3.36 (-1.08, 7.82) | -0.12 (-4.97, 4.72) | 2.12 (-0.57, 4.83) | 0.15 (-3.49, 3.81) | 12.06 (4.48, 19.67)* | 0.68 (-3.24, 4.54) | 1.37 (-1.38, 4.1) | 7.23 (5.24, 9.22)* | 5.76 (1.39, 10.14)* | RT |  |  |
| 1.73 (-0.78, 4.27) | 0.64 (-3.96, 5.21) | 0.6 (-2.21, 3.43) | -0.31 (-2.13, 1.53) | 2.29 (-2.35, 6.93) | -1.19 (-6.2, 3.81) | 1.05 (-1.94, 4.05) | -0.92 (-4.81, 2.98) | 10.99 (3.26, 18.72)* | -0.4 (-4.51, 3.7) | 0.3 (-2.74, 3.32) | 6.15 (3.79, 8.52)* | 4.7 (0.13, 9.27)* | -1.07 (-3.31, 1.15) | SHS |  |
| 1.97 (-0.59, 4.49) | 0.86 (-3.99, 5.72) | 0.83 (-2.41, 4.07) | -0.07 (-2.5, 2.34) | 2.52 (-2.38, 7.43) | -0.96 (-6.24, 4.3) | 1.28 (0.42, 2.15)* | -0.69 (-4.89, 3.52) | 11.22 (3.34, 19.14)* | -0.16 (-4.58, 4.2) | 0.53 (-2.91, 3.95) | 6.38 (3.51, 9.23)* | 4.92 (0.11, 9.73)* | -0.84 (-3.6, 1.89) | 0.24 (-2.82, 3.26) | TWI |

* means p<0.05

B: CK League table

| MD 95%CRI | | | | | | | | | |
| --- | --- | --- | --- | --- | --- | --- | --- | --- | --- |
| ACT |  |  |  |  |  |  |  |  |  |
| -2.04 (-17.03, 13.07) | CON |  |  |  |  |  |  |  |  |
| 90.31 (74.7, 105.96)* | 92.34 (78.31, 106.47)* | CWI |  |  |  |  |  |  |  |
| 569.93 (-213.68, 1358.92) | 572.13 (-210.54, 1360.01) | 479.68 (-302.86, 1266.98) | DO |  |  |  |  |  |  |
| 194.08 (126.78, 261.24)* | 196.13 (130.62, 261.49)* | 103.79 (36.65, 170.64)* | -375.76 (-1164.92, 409.61) | FIR |  |  |  |  |  |
| -90.43 (-190.73, 8.97) | -88.51 (-187.66, 10.05) | -180.81 (-280.95, -81.34)* | -661.29 (-1454.24, 128.85) | -284.51 (-403.77, -166.23)* | Hyp |  |  |  |  |
| 397.46 (183.85, 613.1)* | 399.46 (185.26, 615.44)* | 307.1 (93.11, 523.55)* | -173.38 (-984.14, 639.17) | 203.04 (-20.57, 429.83) | 487.66 (252.37, 725.8)* | INPT |  |  |  |
| -23.89 (-49.33, 1.51) | -21.87 (-42.27, -1.42)* | -114.24 (-138.97, -89.35)* | -594.18 (-1382.3, 188.35) | -217.97 (-286.63, -149.09)* | 66.5 (-33.68, 167.92) | -421.38 (-638.66, -206.27)* | IVO |  |  |
| -102.15 (-522.71, 318.94) | -100.32 (-520.28, 321.09) | -192.5 (-612.67, 229.09) | -671.95 (-1562.51, 217.94) | -296.61 (-722.11, 130.86) | -11.76 (-444.19, 421.82) | -499.83 (-974.69, -25.88)* | -78.4 (-498.45, 343.28) | SHS |  |
| 54.75 (32.98, 76.5)* | 56.8 (36.02, 77.6)* | -35.55 (-50.81, -20.35)* | -515.18 (-1303.01, 268.14) | -139.43 (-207.86, -70.43)* | 145.23 (44.59, 246.5)* | -342.59 (-559.21, -127.91)* | 78.64 (49.41, 107.65)* | 156.78 (-265.07, 577.58) | TWI |

* means p<0.05

C: 20-m sprint League table

| MD 95%CRI | | | | | | |
| --- | --- | --- | --- | --- | --- | --- |
| ACT |  |  |  |  |  |  |
| -0.03 (-10.2, 10.11) | CON |  |  |  |  |  |
| 0.03 (-17.71, 17.62) | 0.06 (-14.31, 14.37) | CWI |  |  |  |  |
| 0.02 (-17.5, 17.64) | 0.07 (-14.25, 14.46) | 0.03 (-20.31, 20.4) | Hyp |  |  |  |
| 7.64 (-10.17, 25.17) | 7.67 (-6.74, 22.09) | 7.59 (-12.72, 28.01) | 7.58 (-12.83, 27.87) | IVO |  |  |
| -0.02 (-14.34, 14.38) | 0.02 (-10.1, 10.17) | -0.05 (-17.38, 17.65) | -0.05 (-17.6, 17.55) | -7.66 (-25.22, 10.04) | RT |  |
| -4.17 (-23.8, 15.14) | -4.12 (-20.85, 12.37) | -4.19 (-12.61, 4.04) | -4.22 (-26.37, 17.7) | -11.79 (-33.95, 10.08) | -4.14 (-23.81, 15) | TWI |

* means p<0.05

D: MVC League table

| MD 95%CRI | | | | | |
| --- | --- | --- | --- | --- | --- |
| ACT |  |  |  |  |  |
| 16.53 (-113.04, 145.77) | CON |  |  |  |  |
| -22.05 (-152.54, 108.16) | -38.43 (-52.63, -24.11)* | FIR |  |  |  |
| -71.74 (-233.32, 89.65) | -88.27 (-184.69, 8.3) | -49.92 (-147.1, 47.6) | Hyp |  |  |
| 0.69 (-128.92, 130.06) | -15.82 (-19.91, -11.7)* | 22.61 (7.75, 37.38)* | 72.52 (-24.16, 168.83) | IVO |  |
| 5.81 (-123.88, 135.24) | -10.7 (-16.27, -5.12)* | 27.72 (12.4, 42.96)* | 77.61 (-19.11, 174.1) | 5.12 (-1.78, 12.05) | PCMcold |

* means p<0.05

E: MS League table

| MD 95%CRI | | | | |
| --- | --- | --- | --- | --- |
| ACT |  |  |  |  |
| 0.25 (-0.16, 0.67) | CON |  |  |  |
| 17.07 (12.57, 21.57)* | 16.82 (12.33, 21.31)* | FIR |  |  |
| 0.53 (-0.35, 1.41) | 0.28 (-0.5, 1.05) | -16.54 (-21.1, -11.98) | Hyp |  |
| 33.18 (15.66, 50.81)* | 32.92 (15.41, 50.54)* | 16.1 (-2.01, 34.31) | 32.65 (15.12, 50.3)* | PCMcold |

* means p<0.05

**Supplementary Table 7.** Comparative effectiveness results for (A) Countermovement jump height (CMJ), (B) Creatine kinase (CK), (C) 20-m sprint performance, (D) Maximal voluntary contraction (MVC), and (E) Muscle soreness (MS). Each cell shows an MD with 95%CrI. CrI = credible interval; MD = mean difference. ACT: Active recovery; atDCS: Transcranial Direct Current Stimulation; CHP: Carbohydrate-protein; CON: Passive recovery; Cre: Creatine; Cryo: Cryo-compression; CWI: Cold-water immersion; Elec: Electrostimulation; FIR: Far-Infrared Radiation; FR: Foam Rolling; Hyp: Hyperoxic gas; IVO: Intermittent Vascular Occlusion; PCMcold: Phase change material(with 15℃); RT: Resistance training; SHS: Sleep Hygiene Strategy; TWI: Thermoneutral water immersion; DO:Deep oscillation; INPT: Intermittent Negative Pressure Therapy

**Supplementary material 6. Ranks of various types of exercise in terms of primary outcomes**

| Treatment | CMJ (%) | CK (%) | 20m-sprint (%) | MVC (%) | MS (%) |
| --- | --- | --- | --- | --- | --- |
| ACT | 66.6 | 36.6 | 47.7 | 43.9 | 5.8 |
| atDCS | 46.4 | NR | NR | NR | NR |
| CHP | 48.1 | NR | NR | NR | NR |
| CON | 31.0 | 34.4 | 47.2 | 8.8 | 28.2 |
| Cre | 65.8 | NR | NR | NR | NR |
| Cryo | 24.6 | NR | NR | NR | NR |
| CWI | 56.4 | 65.9 | 51.2 | NR | NR |
| Elec | 25.1 | NR | NR | NR | NR |
| FIR | 98.3 | 79.1 | NR | 75.7 | 76 |
| FR | 32.5 | NR | NR | NR | NR |
| Hyp | 42.6 | 8.3 | 48.0 | 89.8 | 40.9 |
| IVO | 91.9 | 18.5 | 84.5 | 49.9 | NR |
| PCMcold | 84.2 | NR | NR | 31.9 | 98.9 |
| RT | 17.0 | NR | 47.5 | NR | NR |
| SHS | 37.5 | 23 | NR | NR | NR |
| TWI | 31.9 | 54 | 23.9 | NR | NR |
| DO | NR | 88.2 | NR | NR | NR |
| INPT | NR | 91.9 | NR | NR | NR |

**Supplementary Table 8.** SUCRA (%) for different types of intervention in (A) CMJ, (B) CK, (C) 20-m sprint, (D) MVC, and (E) MS. Higher SUCRA in primary outcomes indicate better-performing interventions. CMJ: Countermovement jump height; CK: Creatine kinase; MVC: Maximal voluntary contraction; MS: Muscle soreness; ACT: Active reco(1)very; atDCS: Transcranial Direct Current Stimulation; CHP: Carbohydrate-protein; CON: Passive recovery; Cre: Creatine; Cryo: Cryo-compression; CWI: Cold-water immersion; Elec: Electrostimulation; FIR: Far-Infrared Radiation; FR: Foam Rolling; Hyp: Hyperoxic gas; IVO: Intermittent Vascular Occlusion; PCMcold: Phase change material(with 15℃); RT: Resistance training; SHS: Sleep Hygiene Strategy; TWI: Thermoneutral water immersion; DO:Deep oscillation; INPT: Intermittent Negative Pressure Therapy; NR: not reported

**Supplementary material 7.**

| Outcomes | Pairwise meta-analysis | No of study | Heterogeneity (%) | MD 95%CrI |
| --- | --- | --- | --- | --- |
| CMJ | CON VS ACT | 2 | 49.9 | -2.09(-7.45, 3.36) |
|  | TWI VS ACT | 1 | NR | -1.55(-9.44, 6.43) |
|  | CON VS atDCS | 1 | NR | -0.93(-9.22, 7.14) |
|  | CON VS CHP | 1 | NR | -0.88(-8.44, 6.63) |
|  | Cre vs CON | 1 | NR | 2.60(-5.59, 10.78) |
|  | Cryo vs CON | 1 | NR | -0.89(-9.21, 7.50) |
|  | CWI vs CON | 1 | NR | 1.09(-6.60, 8.67) |
|  | Elec vs. CON | 1 | NR | -0.60(-8.41, 7.24) |
|  | FIR vs CON | 1 | NR | 11.30(1.06, 21.51) |
|  | FR VS CON | 1 | NR | -0.09(-8.14, 7.82) |
|  | Hyp VS CON | 1 | NR | -0.70(-7.0, 8.10) |
|  | IVO VS CON | 1 | NR | 6.46(-0.83, 13.84) |
|  | PCMcold VS CON | 2 | 75.4 | 5.20(-1.11, 11.82) |
|  | RT VS CON | 2 | 0 | -0.77(-6.02, 4.47) |
|  | SHS VS CON | 1 | NR | 0.29(-7.07, 7.69) |
|  | TWI VS CWI | 3 | 88.5 | -2.09(-7.05, 1.90) |
| Outcomes | Pairwise meta-analysis | No of study | Heterogeneity (%) | MD 95%CrI |
| CK | CON VS ACT | 2 | 0 | -3.63(-110.30, 96.57) |
|  | CWI VS ACT | 1 | NR | -89.10(-234.21, 58.08) |
|  | INPT VS ACT | 1 | NR | -392.72(-651.69, -137.23) |
|  | CWI VS CON | 2 | 0 | -92.75(-231.69, 51.77) |
|  | DO vs CON | 1 | NR | -576.97(-1343.8, 202.22) |
|  | FIR vs CON | 1 | NR | -195.80(-350.73, -38.32) |
|  | Hyp vs CON | 1 | NR | 90.21(-82.16, 259.83) |
|  | IVO vs CON | 1 | NR | 22.13(-362.90, 539.03) |
|  | SHS vs CON | 1 | NR | 94.55(1.49, 5.96) |
|  | TWI VS CWI | 3 | 85.8 | 51.43(-28.36, 143.54) |
| Outcomes | Pairwise meta-analysis | No of study | Heterogeneity (%) | MD 95%CrI |
| MVC | CON VS ACT | 1 | NR | -13.23(-164.29, 129.65) |
|  | FIR VS CON | 2 | 62.7 | 41.95(-14.92, 104.30) |
|  | Hyp VS CON | 1 | NR | 87.39(-31.95, 209.26) |
|  | IVO VS CON | 1 | NR | 15.78 (-65.27, 96.18) |
|  | PCMcold vs CON | 2 | 0 | 12.29(-47.75, 84.97) |
| Outcomes | Pairwise meta-analysis | No of study | Heterogeneity (%) | MD 95%CrI |
| 20m-sprint | CON VS ACT | 1 | 0 | 0.03(-10.16, 10.21) |
|  | CWI VS CON | 1 | NR | -0.09(-14.44, 14.29) |
|  | Hyp VS CON | 1 | NR | -0.09(-14.49, 14.29) |
|  | IVO VS CON | 1 | NR | -7.69(-22.02, 6.70) |
|  | RT vs CON | 2 | 0 | -0.03(-10.18, 10.03) |
|  | TWI vs CWI | 3 | 100 | 4.17(-4.03, 12.67) |
| Outcomes | Pairwise meta-analysis | No of study | Heterogeneity (%) | MD 95%CrI |
| MS | CON VS ACT | 2 | 83.6 | 0.07(-8.44, 8.85) |
|  | FIR VS CON | 2 | 0 | -16.79(-26.15, -7.64) |
|  | Hyp VS CON | 1 | NR | -0.27(-11.88, 11.54) |
|  | PCMcold vs CON | 1 | NR | -33.26(-53.82, -12.45) |

**Supplementary Table 9.**

CMJ: Countermovement jump height; CK: Creatine kinase; MVC: Maximal voluntary contraction; MS: Muscle soreness; ACT: Active recovery; atDCS: Transcranial Direct Current Stimulation; CHP: Carbohydrate-protein; CON: Passive recovery; Cre: Creatine; Cryo: Cryo-compression; CWI: Cold-water immersion; Elec: Electrostimulation; FIR: Far-Infrared Radiation; FR: Foam Rolling; Hyp: Hyperoxic gas; IVO: Intermittent Vascular Occlusion; PCMcold: Phase change material(with 15℃); RT: Resistance training; SHS: Sleep Hygiene Strategy; TWI: Thermoneutral water immersion; DO:Deep oscillation; INPT: Intermittent Negative Pressure Therapy; NR: not reported

**Supplementary material 8. Publication bias**

**
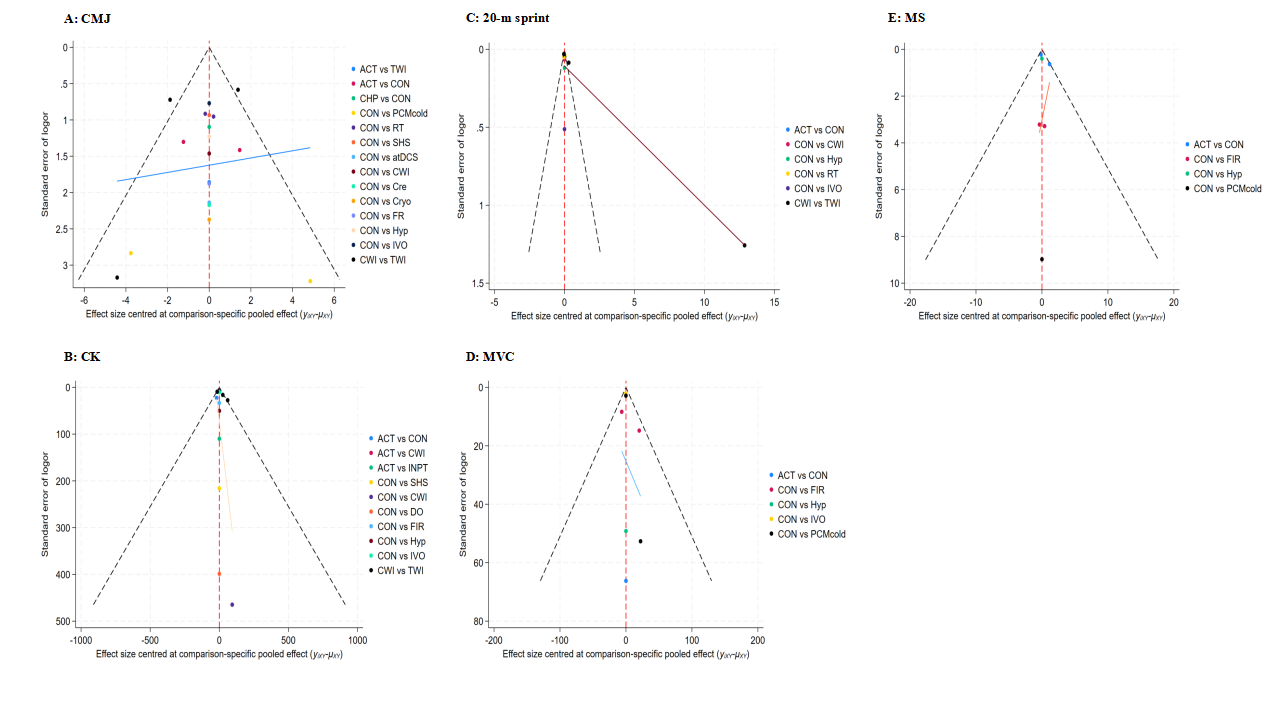
**

**Supplementary Fig. 4.** Publication bias of (A) CMJ, (B) CK, (C) 20-m sprint, (D) MVC, (E) MS

1. Stølen T, Chamari, K., Castagna, C. and Wisløff, U. Physiology of soccer: an update. Sports medicine. 2005.
